# Co-analysis of cucumber rhizosphere metabolites and microbial PLFAs under excessive fertilization in solar greenhouse

**DOI:** 10.3389/fmicb.2022.1004836

**Published:** 2022-10-06

**Authors:** Shuang Wang, Ting Bian, Tong Wu, Yidi Zhang, Muhammad Awais, Hongdan Fu, Zhouping Sun

**Affiliations:** ^1^College of Horticulture, Shenyang Agricultural University, Shenyang, China; ^2^Key Laboratory of Protected Horticulture of the Education Ministry, Liaoning, China; ^3^National and Local Joint Engineering Research Center of Northern Horticultural Facilities Design and Application Technology, Shenyang, China; ^4^Collaborative Innovation Center of Protected Vegetable Surround Bohai Gulf Region, Shenyang, China

**Keywords:** excessive chemical fertilizer, rhizosphere, microbials PLFAs, metabolites, cucumber, solar greenhouse

## Abstract

Fertilizer application is the most common measure in agricultural production, which can promote the productivity of crops such as cucumbers, but the problem of excessive fertilization occurs frequently in solar greenhouses. However, the effects of fertilization levels on cucumber rhizosphere soil microbes and metabolites and their relationships are still unclear. In order to determine how fertilization levels affect the rhizosphere microenvironment, we set up four treatments in the solar greenhouse: no-fertilization (N0P0K0), normal fertilization (N1P1K1), slight excessive fertilization (N2P2K2), and extreme excessive fertilization (N3P3K3). The results showed that fertilization treatments significantly increased cucumber yield compared to no-fertilization, but, the yield of N3P3K3 was significantly lower than that of N1P1K1 and N2P2K2. Fertilization levels had significant effects on rhizosphere microorganisms, and pH, NH_4_^+^-N and AP were the main environmental factors that affected the changes in microbial communities. The total PLFAs, the percentages of fungi and arbuscular mycorrhizal fungi (AMF) were significantly reduced and bacteria percentage was significantly increased in N3P3K3 compared to other fertilization treatments. Differential metabolites under different fertilization levels were mainly organic acids, esters and sugars. Soil phenols with autotoxic effect under fertilization treatments were higher than that of N0P0K0. In addition, compared with soil organic acids and alkanes of N0P0K0, N2P2K2 was significantly increased, and N3P3K3 was not significantly different. This suggested that cucumber could maintain microbial communities by secreting beneficial metabolites under slight excessive fertilization (N2P2K2). But under extremely excessive fertilization (N3P3K3), the self-regulating ability of cucumber plants and rhizosphere soil was insufficient to cope with high salt stress. Furthermore, co-occurrence network showed that 16:1ω5c (AMF) was positively correlated with 2-palmitoylglycerol, hentriacontane, 11-octadecenoic acid, decane,4-methyl- and d-trehalose, and negatively correlated with 9-octadecenoic acid at different fertilization levels. This indicated that the beneficial microorganisms in the cucumber rhizosphere soil promoted with beneficial metabolites and antagonized with harmful metabolites. But with the deepening of overfertilization, the content of beneficial microorganisms and metabolites decreased. The study provided new insights into the interaction of plant rhizosphere soil metabolites and soil microbiomes under the different fertilization levels.

## Introduction

With the constantly increasing of greenhouse area in recent years, greenhouse vegetable cultivation has gradually become an important component and future trend in vegetable production (Zhang et al., 2017). However, some problems have also arisen accordingly. One of them is the overuse of fertilizer by farmers to increase agricultural output (Zhang et al., 2012). China accounts for about 9% of global cropland, but consumes more than 30% of the world’s fertilizers (Chen et al., 2019). Excessive fertilization can lead to soil salinization, acidification and groundwater pollution (Tang et al., 2018; Li et al., 2019; Bindraban et al., 2020; Chynoweth et al., 2021). Muhammad et al. (2008) and Yuan et al. (2007) explained that the accumulation of salinity in soil not only has a direct negative impact on crops. It also has a significant inhibitory effect on microbial respiration, resulting in impaired microbial metabolic function and indirectly affecting crop growth. Inappropriate soil inputs had been reported to disturb the balance between beneficial microbes and metabolites and pathogenic microbes and harmful metabolites (Shen et al., 2010; Chen et al., 2019). It was reported that soil microbial communities and soil metabolites composition were closely related to plant–soil health (Reinhold-Hurek et al., 2015; Dong et al., 2018). Therefore, research on how chemical fertilizer application affects soil microorganisms and metabolites in the rhizosphere is crucial for the long-term sustainability of solar greenhouses.

Soil microbes are key drivers of biogeochemical cycles, energy turnover, and plant growth (Mohanram and Kumar, 2019). Microbial diversity is one of the most important soil health indicators (Wang et al., 2020), it varies with plant, season, soil type, and fertilizer management mode (Garbeva et al., 2004; Zhang et al., 2012; Li et al., 2021). The key responses of soil microbiota to fertilizers can be revealed by analyzing microorganisms (Islam et al., 2011). The 10 year field trial results clearly show that long-term nitrogen fertilization affects soil pH, thereby significantly altering most dominant soil bacterial species (Ren et al., 2020). As well as excessive phosphorus fertilizers reduced microbial diversity and significantly altered microbial community structures and compositions, phosphorus levels also interfered with the complexity of soil bacterial and fungal symbiosis networks (Cheng et al., 2022). Moreover, soil metabolomics provides a window into microbial behavior, as the metabolites secreted by microbes and the residual decomposition products of microbes are the main components of the soil metabolome (Jones et al., 2014). Microbial metabolism includes many simultaneous anabolic and catabolic reactions that inform biochemical activities within the entire soil microbiota (Muhammad et al., 2008; Poeplau et al., 2016). Previous studies had shown that fertilization affects the diversity of soil microbial functions (Shen et al., 2010; Hao et al., 2020), and certain microbial metabolic processes, such as carbohydrate and amino acid metabolism (Li et al., 2021).

In addition to the metabolites secreted by microorganisms and the decomposition of microbial residues, root exudates of plants are also the main components of rhizosphere soil metabolites (Badri and Vivanco, 2009; Song et al., 2020; Smercina et al., 2021). Plant roots secrete a large number of low molecular weight and high molecular weight compounds into the surrounding soil, including sugars, organic acids, amino acids and other secondary metabolites. These rhizosphere sediments account for 10% of the plant net photosynthetically fixed carbon (Mohanram and Kumar, 2019). They are important carbon substrates for soil microorganisms near the roots (Wallace, 1994; Yuan et al., 2016), and as signaling molecules to influence the complex interactions between plants and rhizosphere microbes (Badri and Vivanco, 2009; Mohanram and Kumar, 2019; Phour et al., 2020). Plants can secrete root exudates and change root morphology to influence and recruit the specific microbial communities (Reinhold-Hurek et al., 2015; Dong et al., 2018). It was recently found that rhizosphere fungal community assemblage and species coexistence differ from bulk soils, caused by changes in root exudates under different nitrogen inputs (Wang et al., 2022). Therefore, understanding the effects of fertilizer usage on rhizosphere metabolism is helpful to further understand the effects of fertilizer on soil microbial community and its interactions with plants.

In order to clarify the effect of excessive NPK fertilization on soil microorganisms and metabolites, and to provide a basis for reasonable fertilization of cucumbers in solar greenhouse. In this study, the main cultivated crop in solar greenhouse, cucumber was used as the experimental material, and four fertilization levels were set to conduct pot experiments. The aim was to (1) elucidate the effect of excessive chemical fertilizer application on cucumber rhizosphere soil microbial PLFAs and metabolites in solar greenhouse, and to (2) reveal the interaction between microbial communities and metabolites under different fertilization levels.

## Materials and methods

### Soil preparation and sampling

This experiment was carried out in a solar greenhouse (41°310 N–123°240E) of Shenyang Agricultural University, China. The cucumber variety was “Jinyou 30,” and the soil used in the experiment was garden soil that has never been planted with cucurbit vegetables. The soil chemical properties are as follow: pH = 7.21, soil organic matter (SOM) = 17.8 g kg^−1^, available N (AN) = 105 mg kg^−1^, available P (AP) = 112.8 mg kg^−1^, and available K (AK) = 121.5 mg kg^−1^, were determined by the Bao’s Method (Bao, 2000). According to the national survey and test results of vegetable fertilization (Li et al., 2019). Four fertilization levels were set in the pot experiment (each pot contained 14 kg air-dried soil), including no-fertilization (N0P0K0), normal fertilization (N1P1K1), slight excessive fertilization (N2P2K2), and extreme excessive fertilization (N3P3K3). The four treatments were implemented in a randomized design with five replications. The specific fertilization amounts were shown in Table 1. Except for the fertilization levels, other cultivation and management measures were the same.

**Table 1:** The amount of fertilizer used in the experiment treatments.

Treatment	N (g·plant^−1^)	P_2_O_5_ (g·plant^−1^)	K_2_O (g·plant^−1^)
N0P0K0	0.00	0.00	0.00
N1P1K1	4.30	4.00	4.38
N2P2K2	8.60	8.00	8.75
N3P3K3	12.90	12.00	13.13

The first crop was planted in September 2017, the second in March 2018, and the third in September 2018. In this experiment, samples were collected from the five replicates for each treatment after the third crop. Leaf, stem, and root of cucumber were separated and dried in an oven at 65°C for 48 h until reaching a constant weight for measuring dry weights. The fourth leaf of cucumber was collected for determining photosynthetic pigments according to Lichtenrhaler’s method (Lichtenthaler and Welbum, 1983). Yield was the total fruit fresh weight per plant during cucumber growth. In this study, we took out the entire root system from the pot, after removing the bulk soil around the root system, we use a brush to gently collect the soil attached to the cucumber root. After picking out the plant residues, the soil was sieved through a 2 mm mesh and divided into three subsamples. The first subsample was freeze-dried and stored at −80°C for microbial PLFAs and metabolites measured, the second subsample was placed at 4°C to measure soil enzymes activities, and the third subsamples was air-dried to determine the soil chemical properties.

### Soil chemical properties and enzymes activities determination

Soil pH (soil:H_2_O 1:2.5) was measured with pH meter (sartorius, Germany). SOM was determined by potassium dichromate volumetric method. AP was measured by molybdenum blue spectrophotometry method. AK was determined by atomic adsorption spectrophotometer. The total N (TN) was measured by Kjeldahl method. After the soil digestion with HNO_3_, HCl and HF, total phosphorus (TP) was determined with an ultraviolet spectrophotometer and total potassium (TK) was determined with atomic absorption flame spectrophotometer (Bao, 2000). NH_4_^+^-N and NO_3_^−^-N were extracted with 1 mol·L^−1^ KCl and measured using a continuous colorimetric flow system (Skalar SAN++ system, Netherlands) (Zhou et al., 2010). Urease was measured by indophenol blue colorimetric method. Polyphenol oxidase was determined by iodine titration and neutral phosphatase activities were determined by phenyl disodium phosphate colorimetry (Guan, 1986; Zhang et al., 2011).

### PLFA analysis

The microbial PLFAs were measured according to previous methods (Bossio, 1998; Briar et al., 2011). Briefly, lipids were extracted from 8 g of freeze-dried soil using a mixed extract (phosphate buffer:chloroform:methanol mixture, 0.8:1:2, v/v/v), and separated PLFAs from neutral lipids and glycolipids fatty acids using a SPE silica column (Supelco Inc, Bellefonte, PA). Using 19:0 methyl ester as internal standard. PLFAs were measured using gas chromatograph (Agilent 6850 Series, USA) equipped with the Sherlock microbial identification system (MIDI Inc., Newark, USA). Specific PLFA fingers could represent specific microbial communities, and the classification results of PLFAs were shown in Supplementary Table S1 (McKinley et al., 2005; Aciego Pietri and Brookes, 2009; Bach et al., 2010).

### Soil metabolite profiling analysis

The soil metabolite extraction method was modified according to Song et al. (2020). Briefly, 1 g freeze-dried soil was placed in 5 ml Eppendorf tube, added 1 ml methanol:H_2_O = 3:1 (v/v), 1 ml ethyl acetate, and 10 μl 0.5 mg ml^−1^ adonitol dissolved in deionized water (internal standard) sequentially. Samples were homogenized at 45 Hz for 4 min and sonicated in ice water for 5 min. Then centrifuged at 12000r for 15 min at 4°C. All the supernatants were dried completely *in vacuo* at 28°C, and then dissolved in 60 μl of methoxylamine hydrochloride (15 mg ml^−1^ in pyridine) followed by 30 min of incubation at 80°C. Then, 80 μl of BSTFA (with 1% TMCS) was added to each sample and incubated for 90 min at 70°C. Finally, The derivatized sample was analyzed using GC–MS (7890A-5795C, Agilent, Palo Alto, Calif., USA) system equipped with Agilent HP-5MS column (30 m × 250 μm × 0.25 μm). The injection volume was 1 μl, the helium gas flow rate was 1 ml min^−1^. The injector temperature and ion source temperature were at 250 and 230°C, respectively. The oven program was as follows: the initial temperature was kept at 50°C for 2 min, raised to 120°C at a rate of 5°C min^−1^, then hold for 2 min at 120°C, increased to 180°C at a rate of 6°C min^−1^ and hold for 2 min, and finally increased to 250°C at a rate of 4°C min^−1^ and hold for 12 min.

The quality control (QC) sample was prepared by pooling 10 μl aliquots of all samples to check the stability of the instrument throughout the analysis. Compound identification was performed by mass comparison with NIST 08 (http://www.nist.gov) mass spectrometry library. The detailed classification information of metabolites was shown in Supplementary Table S2.

### Statistical analysis

Significant differences in plant growth parameters, soil chemical properties, soil enzymes activities, soil microbial PLFAs and relative abundance of classified soil metabolites among fertilization treatments were examined using one-way analysis of variance (ANOVA) and mean values were compared with Duncan’s multiple range test (*p* < 0.05) using the SPSS version 19.0 software package (SPSS, Inc., Chicago, IL, United States). The histograms were drawn using origin 2019b. The partial least-square discriminant analysis (PLS-DA) based on metabolomics data were performed using SMICA-P software (version 14.0). The differential metabolites were selected by a variable importance (VIP) > 1 and *p* < 0.05 (Jia et al., 2019). Using vegan (Oksanen et al., 2013) and ggplot2 (Wickham, 2011) packages to draw redundancy analysis (RDA) and heatmap based on microbial PLFAs data in R v.4.1.0. The correlation and visualization between microbial PLFAs and differential metabolites were performed using R and Cytoscape 3.7.2 software.

## Results

### Plant growth

**Figure 1:**
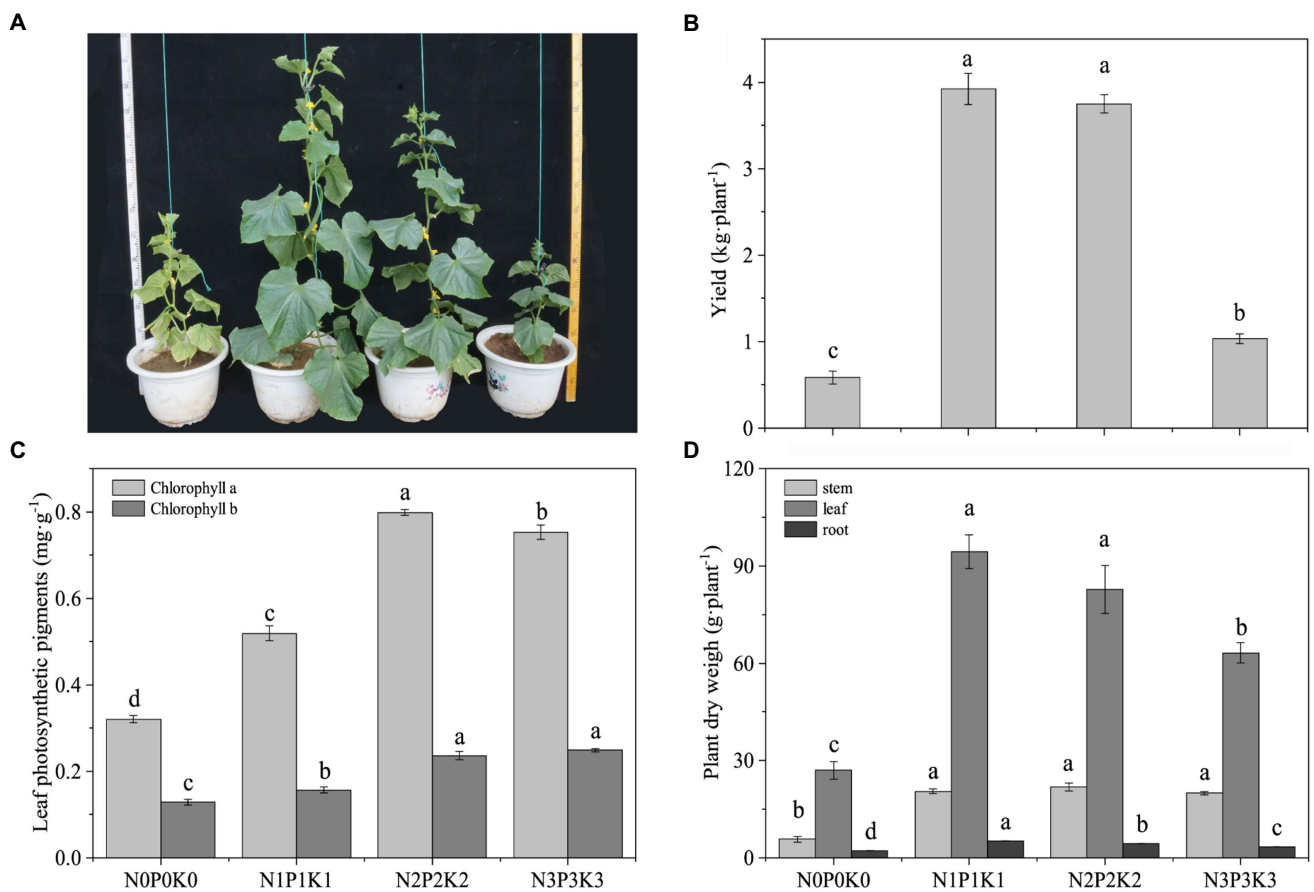
The effects of different fertilization levels on **(A)** plant images, **(B)** cucumber yields, **(C)** leaf photosynthetic pigments, and **(D)** plant dry weights. Values are means ± SEM (*n* = 5). Different letters above the bars indicate statistically significant differences at *p* < 0.05.

Different fertilization levels had an obvious effects on cucumber plant morphology (Figure 1A). Compared with no-fertilization, fertilization led to a significant increase in cucumber yield, and the yield of N1P1K1 and N2P2K2 were significantly higher than that of N3P3K3 (Figure 1B). Fertilization significantly increased the chlorophyll contents in leaves, and the chlorophyll a under N2P2K2 treatment and the chlorophyll b under the N2P2K2 and N3P3K3 treatments were significantly higher than those under the other treatments (Figure 1C). In addition, the plants dry weights under fertilization treatments were significantly higher than those under no-fertilization treatment. However, from N1P1K1 to N3P3K3 treatments, leaf and root dry weights decreased significantly overall (Figure 1D).

### Soil chemical properties and enzymes activities

Soil pH decreased significantly, and contents of NH_4_^+^-N, NO_3_^−^-N, AP, AK, TN, TP and TK significantly increased with the increase of chemical fertilization. In addition, the SOM contents under N3P3K3 treatment were significantly higher than those under the N0P0K0 and N1P1K1 treatments (Table 2).

**Table 2:** The effects of different fertilization levels on soil chemical properties.

Treatment	pH	NH_4_^+^-N	NO_3_^−^-N	AP	AK	TN	TP	TK	SOM
		(mg·kg^−1^)	(mg·kg^−1^)	(mg·kg^−1^)	(mg·kg^−1^)	(g·kg^−1^)	(g·kg^−1^)	(g·kg^−1^)	(g·kg^−1^)
N0P0K0	7.31 ± 0.01a	4.99 ± 0.23c	4.99 ± 0.56d	39.63 ± 0.92d	106.47 ± 5.54c	1.39 ± 0.01b	0.69 ± 0.05d	14.96 ± 0.21c	16.29 ± 0.06b
N1P1K1	7.19 ± 0.02b	9.06 ± 1.16bc	47.26 ± 5.20c	84.42 ± 2.29c	138.73 ± 4.53c	1.39 ± 0.02b	1.11 ± 0.09c	17.36 ± 0.36ab	16.21 ± 0.19b
N2P2K2	7.09 ± 0.04c	12.20 ± 1.74b	142.14 ± 6.64b	129.38 ± 4.86b	269.95 ± 22.68b	1.40 ± 0.01b	1.36 ± 0.02b	16.95 ± 0.48b	16.39 ± 0.13ab
N3P3K3	6.81 ± 0.04d	35.54 ± 1.80a	169.64 ± 6.73a	162.27 ± 5.82a	339.17 ± 19.44a	1.52 ± 0.01a	1.74 ± 0.05a	18.37 ± 0.34a	16.78 ± 0.10a

Values are means ± SEM (*n* = 5). Different letters between all treatments indicate statistically significant differences at *p* < 0.05.

**Figure 2:**
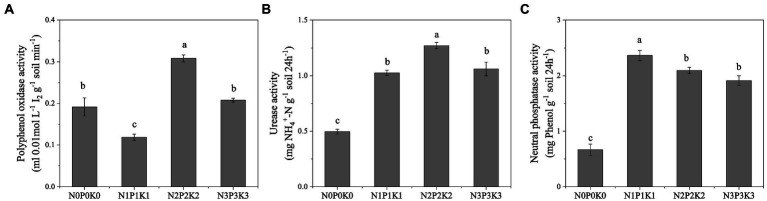
The effects of different fertilization levels on soil enzymes activities. Values are means ± SEM (*n* = 5). Different letters above the bars indicate statistically significant differences at *p* < 0.05.

The effect of different fertilization levels on soil enzyme activities were shown in Figure 2. The activity of polyphenol oxidase decreased first and then increased with the fertilization rates. Among the four treatments, the activity of polyphenol oxidase in N1P1K1 was the lowest and that in N2P2K2 was the highest (Figure 2A). With the increase of fertilization, the activities of urease and neutral phosphatase both increased first and then decreased significantly, and the highest points of enzyme activities were N2P2K2 and N1P1K1, respectively (Figures 2B,C).

### Soil microbial community structure

**Figure 3:**
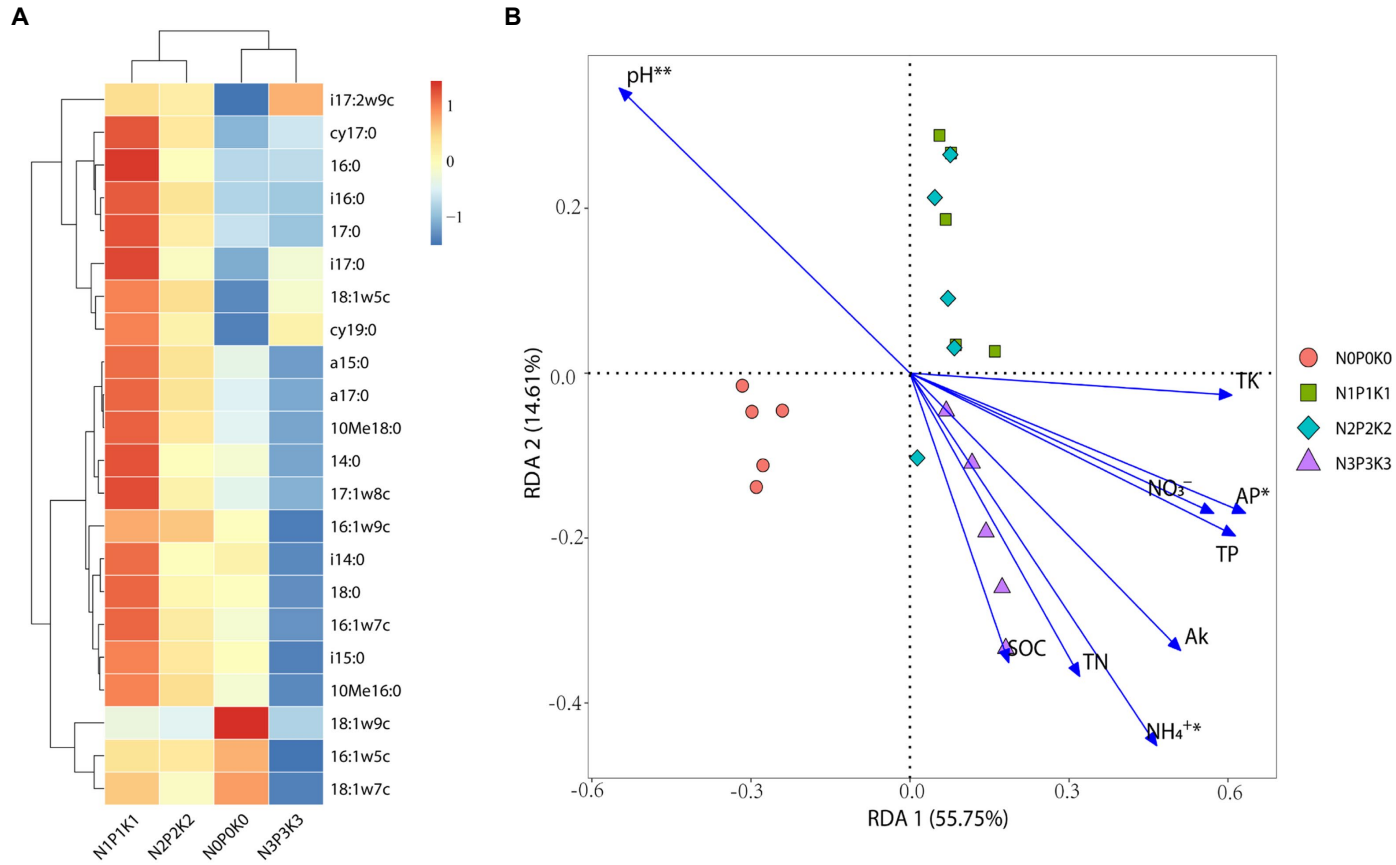
The effects of different fertilization levels on soil microbial communities. **(A)** Heatmap of relative abundance of microbial PLFAs, reddish color indicates increased metabolites content while bluish color indicates decreased metabolites content. **(B)** Redundancy analysis (RDA) of the rhizosphere microbial PLFAs with soil chemical properties, Significant variables *via* forward selection are labeled with asterisk (**, and * represent *p* < 0.01, and 0.05, respectively).

In this study, total PLFAs first increased and then decreased with fertilization rate, and among the four fertilization treatments, total PLFA was significantly highest under N1P1K1 and lowest under N3P3K3 (Table 3). The percentage of bacteria was significantly higher under N3P3K3 treatment than other treatments. The percentages of fungi and arbuscular mycorrhizal fungi (AMF) were significantly lower under N3P3K3 treatment than other treatments. In addition, the percentages of actinomycetes under N1P1K1 and N2P2K2 treatments were significantly higher than those under N0P0K0 and N3P3K3 treatments (Table 3). A total of 22 PLFAs were detected in rhizosphere soils under different fertilization gradients (Figure 3A). Amongst them, the relative abundance Gram-negative bacteria (16:0, 18:1ω7c, 18:1ω5c and 16:1ω7c), Gram-positive bacteria (i15:0, i17:2ω9c, cy19:0, a15:0 and i16: 0), actinomycetes (10Me16:0), and fungi (18:1ω9c, 16:1ω5c) were all more than 3%, and they accounted for 82.67–83.17% of the total PLFAs. Notably, the relative abundances of 16:1ω5c, 18:1ω7c and 18:1ω9c decreased with increasing fertilizer application (Figure 3A).

**Table 3:** The effects of different fertilization levels on soil total PLFA and percentage of microbial PLFAs.

Treatment	Total PLFA (nmol·g^−1^ soil)	Bacteria (%)	Gm^+^ (%)	Gm^−^ (%)	Fungi (%)	AMF (%)	Actinomycetes (%)
N0P0K0	125.13 ± 3.05b	73.35 ± 1.55b	41.95 ± 3.07b	31.39 ± 1.76ab	6.62 ± 0.48a	3.95 ± 0.36a	13.15 ± 0.65b
N1P1K1	143.08 ± 3.37a	72.20 ± 0.72b	39.04 ± 0.54b	33.16 ± .79a	6.63 ± 0.23a	3.54 ± 0.22a	15.23 ± 0.37a
N2P2K2	120.55 ± 4.63c	71.62 ± 0.41b	39.57 ± 0.49b	32.05 ± 0.45ab	6.56 ± 0.12a	3.65 ± 0.14a	15.89 ± 0.21a
N3P3K3	96.14 ± 7.99d	76.92 ± 1.63a	48.27 ± 3.01a	28.65 ± 1.44b	4.66 ± 0.42b	2.19 ± 0.35b	12.54 ± 1.09b

Values are means ± SEM (*n* = 5). Different letters between all treatments indicate statistically significant differences at *p* < 0.05. AMF: arbuscular mycorrhizal fungi.

RDA showed that no-fertilization and fertilization treatments were clearly distinguished along the first axis with described 55.75% of variations in the microbial PLFAs. Furthermore, the microbial PLFAs of N1P1K1 and N2P2K2 treatments were clustered together, and along the second axis which described 14.61% of variations significantly distinguished with the N3P3K3 treatment (Figure 3B). With the increment of fertilization, the pH (*p* = 0.001) decreased significantly, the NH_4_^+^-N (*p* = 0.0464) and AP (*p* = 0.016) increased significantly, which had the obviously effects on the changes of microbial communities (Figure 3B).

### Soil metabolites

**Figure 4:**
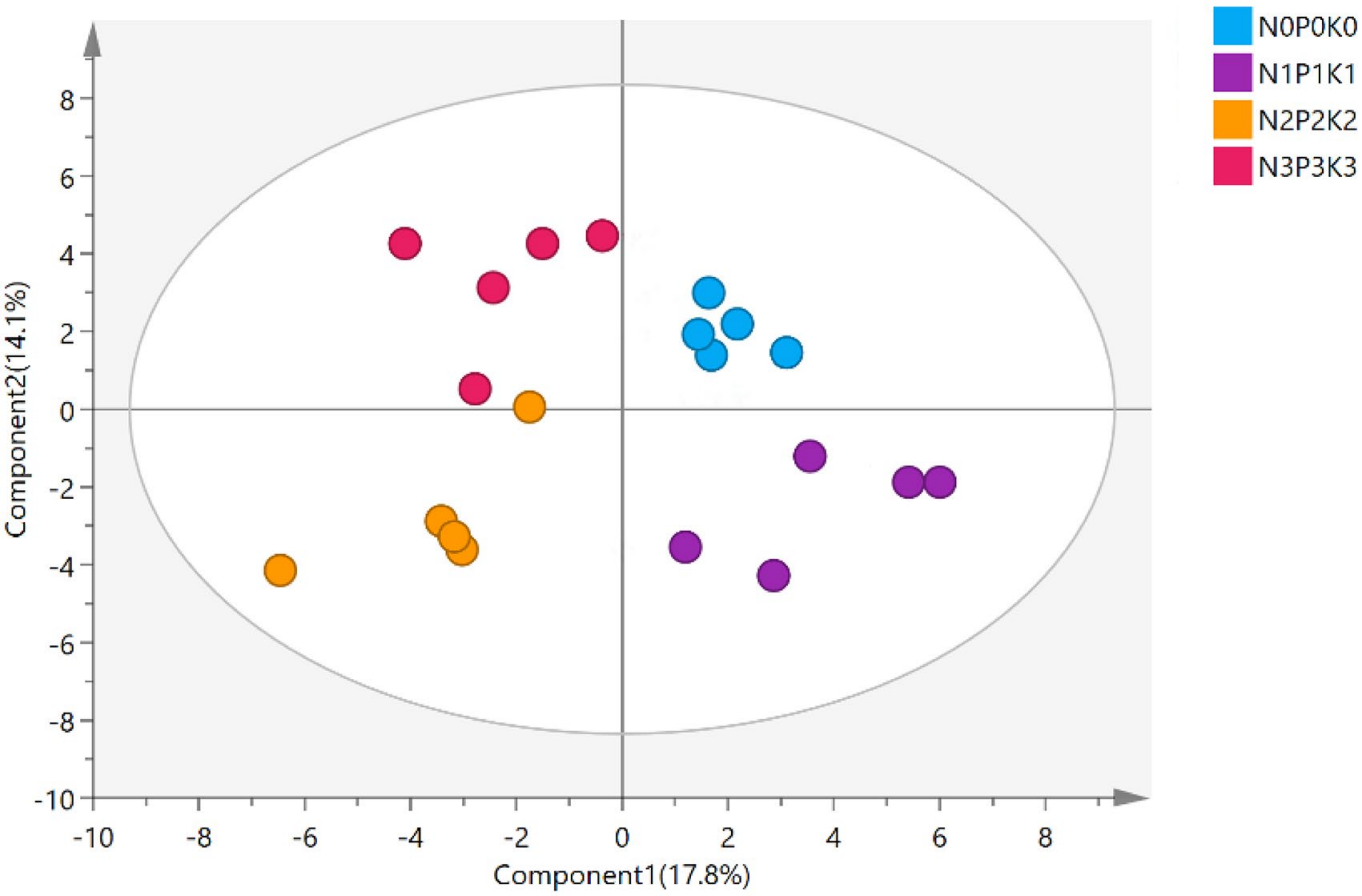
The partial least squares-discriminant analysis (PLS-DA) of soil metabolites under different fertilization levels.

**Figure 5:**
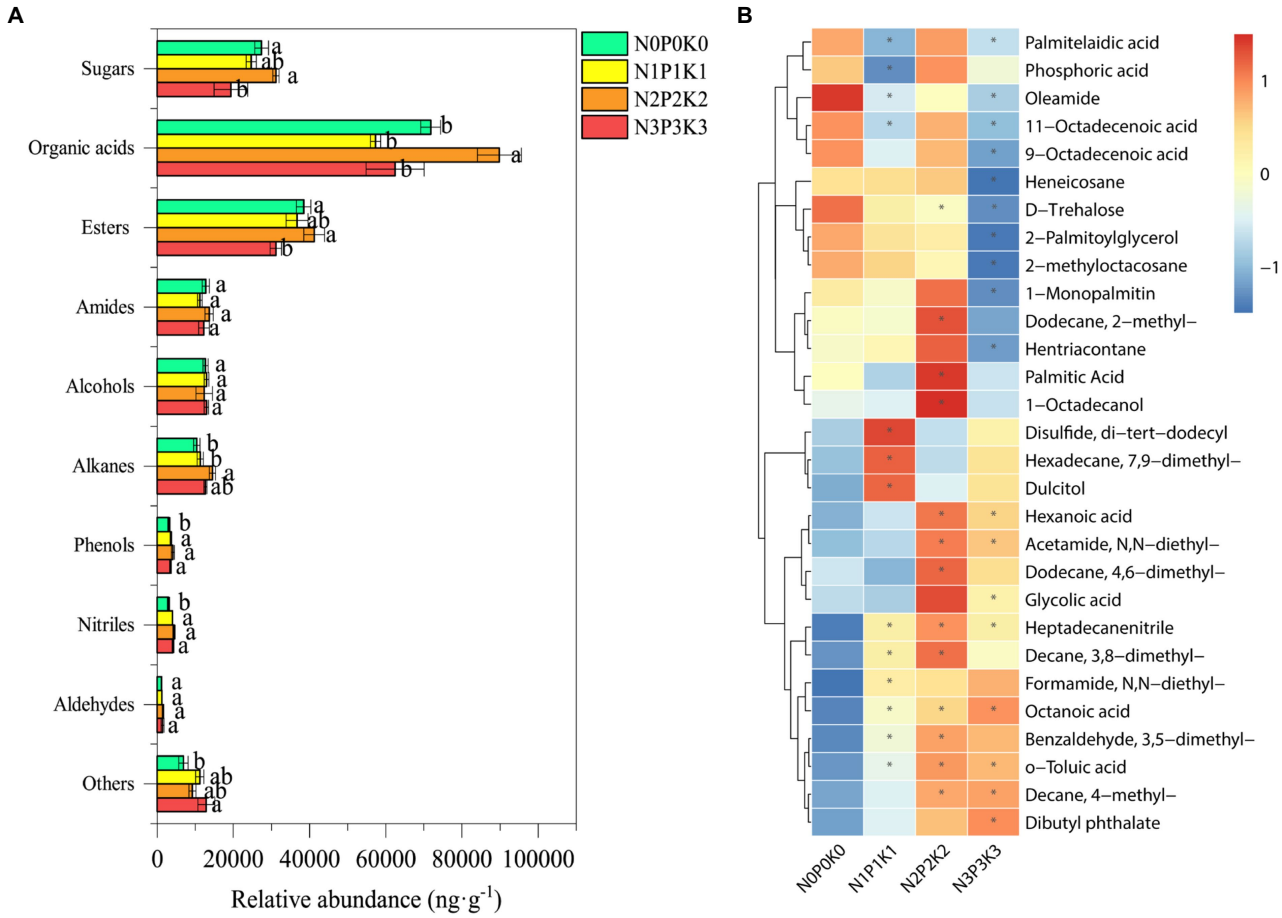
**(A)** Relative abundance of classified metabolites under different fertilization levels. Values are means ± SEM (*n* = 5). Different letters above the bars indicate statistically significant differences at *p* < 0.05. **(B)** Heatmap of differential metabolites under different fertilization levels, reddish color indicates increased metabolites content while bluish color indicates decreased metabolites content, * represent differential metabolites for three fertilization treatments compared to no-fertilization (VIP > 1 and *p* < 0.05).

A total of 69 metabolites were detected and identified in all treatments, the specific information of soil metabolites listed in Supplementary Table S2. The PLS-DA score plot showed the soil metabolites under four fertilization levels were clearly distinguished, the first and second axis, respectively, described 17.8% and 14.1% of variations in the metabolites (Figure 4), Metabolites were divided into 10 categories according to their molecular structures (Figure 5A). Soil sugars and esters contents were significantly lowest under N3P3K3 treatment than other treatments. The content of organic acids of N2P2K2 treatment was significantly higher than those of N0P0K0, N1P1K1 and N3P3K3 treatments. The content of alkanes under N2P2K2 treatment was significantly higher than those under N0P0K0 and N1P1K1 treatments. In addition, phenols and nitriles contents were significantly higher under fertilization treatments than N0P0K0 treatment, and others content were significantly higher under N3P3K3 treatment compared to N0P0K0 treatment (Figure 5A).

The relative abundance of differential metabolites among four fertilization treatments were shown in heatmap (Figure 5B). In our study, the main differential metabolites were organic acids, esters, sugars, etc. Specifically, the relative abundances of formamide, N,N-diethyl-, octanoic acid, decane,4-methyl- and dibutyl phthalate were gradually increased, while the relative abundances of d-trehalose, 2-palmitoylglycerol and 2-methyloctacosane were gradually down-regulated with fertilization addition rates (Figure 5B).

### Correlations between the soil metabolism and microbial PLFAs

**Figure 6:**
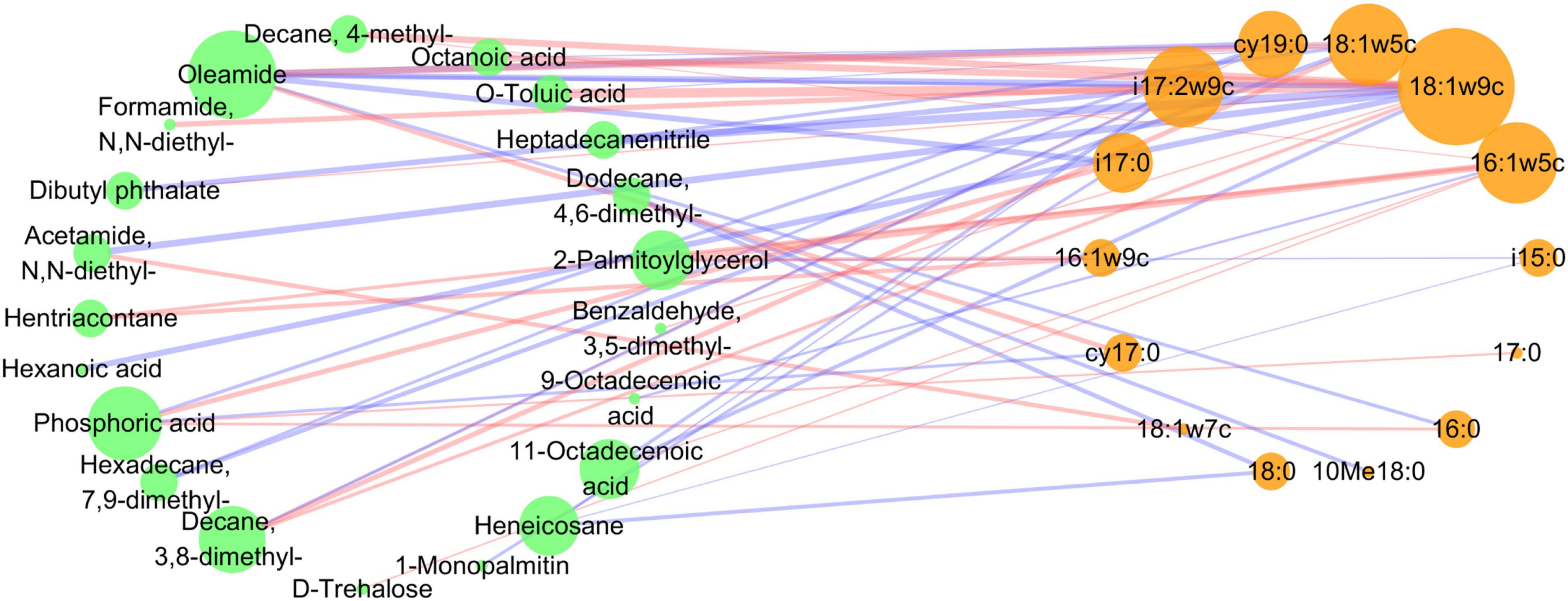
Co-occurrence network of the microbial PLFAs and differential metabolites under different fertilization levels. A connection indicates a strong correlation (spearman correlation analysis, *p* < 0.05), the stronger the spearman correlation, the thicker the line. The size of each node is proportional to the number of connections, the blue and red lines indicate negative and positive relationships, respectively.

To further elucidate the relationship between microbial PLFAs and metabolites under different fertilization levels, a correlation network was constructed (Figure 6). There were more negative correlations than positive correlations between soil microbial PLFAs and differential metabolites (22 positive correlations and 27 negative correlations). The most relevant metabolites with microbial PLFAs were oleamide, phosphoric acid and decane,3,8-dimethyl. The microbial PLFAs closely related to metabolites were 18:1ω9c, 18:1ω5c, 16:1ω5c and i17:2ω9c. Specifically, 18:1ω9c was positively correlated with decane,3,8-dimethyl-, 11-octadecenoic acid, benzaldehyde,3,5-dimethyl- and dibutyl phthalate, and negatively correlated with heptadecanenitrile, O-toluic acid, octanoic acid, decane,4-methyl-, acetamide,N,N-diethyl-, oleamide and hexanoic acid. 18:1ω5c was positively correlated with heptadecanenitrile, O-toluic acid and octanoic acid, and negatively correlated with oleamide, hexadecane,7,9-dimethyl- and decane,3,8-dimethyl-. i17:2ω9c was positively correlated with 11-octadecenoic acid and heneicosane, and negatively correlated with formamide,N,N-diethyl-, dibutyl phthalate, oleamide and decane,3,8-dimethyl-. And 16:1ω5c was positively correlated with 2-palmitoylglycerol, hentriacontane, 11-octadecenoic acid, decane,4-methyl- and d-trehalose, and negatively correlated with 9-octadecenoic acid.

## Discussion

Appropriate nutrient inputs in crop production systems can help to increase crop yields. It has been reported that at least 30 to 50% of crop yield can be attributed to commercial fertilizer nutrient inputs (Stewart et al., 2005). In this study, the yields of N1P1K1 and N2P2K2 were significantly increased by 5.69- and 5.40-fold compared to N0P0K0, respectively (Figure 1). Chemical fertilizers are frequently applied in much bigger amounts than crops are actually consumed to improve crop yields. This resulted in excess of N, P and K in the soil, which could cause soil obstacles and affect crop productivity (Dong and Lin, 2020). The results of this study are consistent with this, the cucumber yield of N3P3K3 was much lower than those of N1P1K1 and N2P2K2 (Figure 1).

Excessive fertilization led to soil nutrient accumulation and soil acidification (Table 2). In addition, polyphenol oxidase activity of N2P2K2 was significantly higher than other fertilization treatments (Figure 2A). Polyphenol oxidase is common redox enzyme related to decomposing toxic substances in soil, and plays a role in soil environmental remediation (Fioretto et al., 2000; Borowik et al., 2014; Grosso et al., 2016). It was reported that in addition to adversely affecting the soil, excessive fertilization could stimulate some self-healing abilities of soil, but this ability decreased with the severity of excessive fertilization. Urease and phosphatase were common indicators that were used to evaluate the ability of microorganisms to utilize nitrogen and phosphorus (Adetunji et al., 2017). The urease and neutral phosphatase activities under fertilization treatments were significantly higher than under N0P0K0 treatment (Figures 2B,C), this might be caused by the low soil nutrient content of N0P0K0 treatment. However, the activities of urease and neutral phosphatase of N3P3K3 were significantly lower than those of N2P2K2 and N1P1K1 treatments, indicating that the soil nutrient utilization ability would decrease under excessive fertilization.

Fertilizer addition had a significant effect on rhizosphere microbial PLFAs (Figure 3A). Total PLFAs of N3P3K3 were significantly lower than other treatments (Table 3). This might be related to soil acidification and nitrate accumulation caused by excessive fertilization (Table 2; Figure 3B). It was consistent with previous studies that soil acidification and high salinity stress could negatively impact the composition and function of soil microbial communities, leading to the attenuation of microbial biomass (He et al., 2007; Kamble et al., 2014; Wei et al., 2017; Becker et al., 2020). The majority of bacteria were better suited to nutrient-rich and highly available carbon sources, while fungus appeared to be more capable of utilizing stubborn carbon sources (He et al., 2007; Grosso et al., 2016). Accordingly, as fertilizer input increased, the percentage of bacteria increased, while the percentage of fungi decreased (Table 3). In addition, the percentage of AMF was significantly lower under N3P3K3 treatment than other treatments (Table 3). AMF is a ubiquitous soil microorganism that can form a mutualistic symbiotic relationship with plant roots, transferring immobile phosphorus resources from great distances to the vicinity of roots (Lin et al., 2012). However, in the case of nutrient sufficiency or even overuse, plants do not need to rely on AMF symbiosis to obtain nutrients, so the relative abundance of AMF decreases with the amount of fertilization (Table 3). In the study, the relative abundances of 16:1ω5c, 18:1ω7c, 18:1ω9c decreased with increasing fertilization rates, suggesting that they could serve as main microbial markers sensitive to excessive fertilization (Figure 3A). Similarly, 18:1ω7c (gram-negative bacteria) has been reported to respond negatively to nitrogen addition (Liu et al., 2015).

Soil metabolites are substances produced by plant photosynthesis, secreted by roots into the rhizosphere soil, and transformed by soil microbial communities. Metabolites play important roles in regulating both plant growth and soil microbial communities (Son et al., 2016; Lu et al., 2022). Previous studies have shown that different fertilization levels have significant effects on soil rhizosphere metabolite profiles (Figure 5). Fertilization affects the root morphology and root exudates of grassland plants to improve plant fitness (Lemanski and Scheu, 2014). Phenols are generally considered to be highly active autotoxins. It has been reported that the accumulation of phenols in soil can inhibit the growth and development of plants, which are among the key reasons for continuous cropping obstacles (Deng et al., 2017; Wu et al., 2017). In the study, soil phenol contents were higher under fertilization treatments than that under no-fertilization treatment (Figure 5A), elucidating that fertilizer inputs might accelerate the progression of continuous cropping obstacles. In addition, compared with soil organic acids and alkanes of N0P0K0, N2P2K2 was significantly increased, and N3P3K3 was not significantly different (Figure 5A). Previous report suggested that the secretion of organic acids might be a strategy for plants to recruit beneficial microorganisms in the root zone in response to high N input (Chen et al., 2019). Cucumbers tend to increase disease resistance by secreting more organic acids to aggregate beneficial microorganisms (Wen et al., 2020). Alkanes could fight microbial pathogens and inhibit the reproduction of pathogenic bacteria (Lu et al., 2022). Plants adapt to biotic and abiotic stresses by altering their root-secreted chemicals to aggregate health-promoting microbiomes. This so-called “cry-for-help” hypothesis provides an explanation for the feedback response of rhizosphere soils to stress (Rolfe et al., 2019). Therefore, it was speculated that organic acids and alkanes might play a key role in recruiting rhizosphere growth-promoting bacteria (PGPR) and affecting the microbial communities to alleviate the stress of slight excessive fertilization (N2P2K2). However, in extreme excessive fertilization (N3P3K3), the self-regulation of plants and soils might no longer be able to cope with high salinity stress. Because of high fertilizer input, the underdeveloped root system of crops communicates less efficiently with soil microbial communities. And in the case of poor soil quality, beneficial metabolites in the rhizosphere soil are more likely to be hijacked by parasitic microorganisms and arthropods (Rolfe et al., 2019). In addition, it was worth noting that the content of dibutyl phthalate (DBP) was up-regulated and the content of d-trehalose was down-regulated with fertilizer addition levels (Figure 5B). DBP might threaten the stability of soil microbial communities and functions, sustainable development of agriculture and human health (Xu et al., 2008). Extensive application of chemical fertilizers and pesticides in agriculture might lead to the accumulation of DBP in soil (He et al., 2015). Trehalose as a protective agent could positively affect plant growth and overcome the adverse effects of salt stress (Jain and Roy, 2009; Sadak et al., 2019). However, with the increase of fertilizer input, trehalose content also decreased, which might be related to the decrease in the abundance of nitrogen-fixing microorganisms capable of synthesize trehalose with higher fertilizer application (Garg and Chandel, 2011).

The correlation between rhizosphere microorganisms and metabolites is very important for the homeostasis of soil environment. In the study, it was found that there were more negative correlations than positive correlations between microorganisms and differential metabolites under different fertilization levels (Figure 6). Because rhizosphere metabolites are the most easily consumed and utilized carbon sources by microorganisms (Yuan et al., 2016). In this study, 18:1ω9c, 18:1ω5c, 16:1ω5c and i17:2ω9c in rhizosphere microbial PLFAs were more closely related to metabolites under different fertilization levels (Figure 6). Notably, 16:1ω5c (AMF) is a beneficial microorganism that forms a symbiotic relationship with plant roots (Lin et al., 2012). Its relative abundance decreased significantly with increasing fertilizer inputs (Figure 3A). In this study, AMF was positively correlated with esters (2-palmitoylglycerol), alkanes (hentriacontane, decane,4-methyl-), organic acids (11-octadecenoic acid) and sugars (d-trehalose), and negatively correlated with organic acids (9-octadecenoic acid) (Figure 6). It has been reported that plant esters are most likely transferred from host plants to AMF in the form of 2-palmitoylglycerol (Kameoka et al., 2019), so 2-palmitoylglycerol was significantly positively correlated with AMF. Alkanes was known to be strong resistant to plant pathogens (Lu et al., 2022). 11-Octadecenoic acid has broad-spectrum antibacterial activity, showing dose-dependent antioxidant activity (Alqahtani et al., 2019). And sugars were not only carbon source that utilized by microorganisms, but also modulates the chemotaxis of rhizosphere bacteria (Eilers et al., 2010; Jin et al., 2019). In addition, 9-octadecenoic acid, which was significantly negatively correlated with AMF, had been shown to be autotoxin, affecting germination and seedling growth in wheat and Sinapis arvensis (Tahir et al., 2018). This indicated that with the increase of fertilization amount, the contents of beneficial microorganisms and metabolites in the rhizosphere soil decreased. Excessive fertilization had an adverse effect on the rhizosphere soil environment.

## Conclusion

Excessive fertilization led to enrichment of soil nutrients and soil pH decreased. Compared with other treatments, total PLFAs content, arbuscular mycorrhizal fungi (AMF) content and fungi percentage were significantly decreased, while percentage of bacteria was significantly increased under N3P3K3. Compared with soil organic acids and alkanes of N0P0K0, N2P2K2 was significantly increased, and N3P3K3 was not significantly different. In addition, the yield of N1P1K1 and N2P2K2 were significantly higher than that of N3P3K3. This suggested that the fertilization of N1P1K1 was more reasonable, as N2P2K2 did not significantly improve yield. The non-decreased yield of N2P2K2 might be due to the fact that cucumber maintained normal growth and yield by secreting beneficial metabolites (organic acids and alkanes) to alleviate the adverse effects on microorganisms of slight excessive fertilization. The significant decrease in N3P3K3 yield was closely related to the decrease of beneficial microorganisms and metabolites in the rhizosphere soil with the amount of fertilization. These results not only clarified the effect of excessive NPK fertilization on soil microorganisms and metabolites, but also provided a theoretical basis for guiding the rational use of chemical fertilizers and promoting the sustainable development of cucumber production in solar greenhouse.

## Data availability statement

The original contributions presented in the study are included in the article/Supplementary material, further inquiries can be directed to the corresponding authors.

## Author contributions

SW and TB: data curation. ZS and HF: funding acquisition. SW, TB, TW, and YZ: experiments. SW: writing-original draft. ZS, HF, and MA: writing-review and editing. All authors contributed to the article and approved the submitted version.

## Funding

This study was financially supported by the National Key R&D Program of China (2020YFD1000301),China Agriculture Research System (CARS-23-D01), the National Natural Science Foundation of China (31902093) and Shenyang Science and technology project (21109308).

## Conflict of interest

The authors declare that the research was conducted in the absence of any commercial or financial relationships that could be construed as a potential conflict of interest.

## Publisher’s note

All claims expressed in this article are solely those of the authors and do not necessarily represent those of their affiliated organizations, or those of the publisher, the editors and the reviewers. Any product that may be evaluated in this article, or claim that may be made by its manufacturer, is not guaranteed or endorsed by the publisher.
