# Supplementary material for: Co-analysis of cucumber rhizosphere metabolites and microbial PLFAs under excessive fertilization in solar greenhouse
Source: Front Microbiol. 2022 Oct 6;13:1004836. doi: 10.3389/fmicb.2022.1004836 (PMC9582138; doi:10.3389/fmicb.2022.1004836)
Supplement: Supplementary file 1 [file Table_2.DOCX]

Supplementary Material

# Supplementary Table

**Supplementary Table S1** The classification of the microbial PLFAs

| Microbial group | Specific PLFA markers |
| --- | --- |
| Gram-positive bacteria (G-) | i14:0, i15:0, a15:0, i16:0, i17:0, i17:2ω9c, a17:0 |
| Gram-negative bacteria (G+) | 16:1ω9c, 16:1ω7c, cy17:0, 17:1ω8c, 18:1ω5c, 18:1ω7c, cy19:0, 14:0, 16:0, 17:0, 18:0 |
| Fungi | 16:1ω5c (AMF), 18: 1ω9c |
| Actinomycetes | 10Me16:0, 10Me18:0 |

**Supplementary Table S2** Summary of detected soil metabolites by class and retention time (RT, min).

| RT | Metabolites | Class | | RT | Metabolites | | Class | |
| --- | --- | --- | --- | --- | --- | --- | --- | --- |
| 12.18 | Lactic Acid | organic acid | | 10.361 | Decane, 4-methyl- | | alkanes | |
| 12.364 | Hexanoic acid | | | 16.375 | Undecane, 2,6-dimethyl- | | | |
| 12.547 | Glycolic acid | | | 18.869 | Dodecane, 4,6-dimethyl- | | | |
| 15.007 | (R)-3-Hydroxybutyric acid | | | 21.29 | Dodecane, 2,6,11-trimethyl- | | | |
| 18.028 | Octanoic acid | | | 22.514 | Dodecane, 2-methyl-6-propyl- | | | |
| 21.072 | O-Toluic acid | | | 22.572 | 3,5-Dimethyldodecane | | | |
| 29.713 | Dodecanoic acid | | | 23.624 | Hexadecane, 7,9-dimethyl- | | | |
| 34.897 | Myristic acid | | | 24.042 | Tridecane | | | |
| 37.311 | Pentadecanoic acid | | | 24.116 | Dodecane, 2-methyl- | | | |
| 39.394 | Palmitelaidic acid | | | 24.219 | Decane, 3,8-dimethyl- | | | |
| 39.806 | Palmitic Acid | | | 24.414 | Dodecane | | | |
| 41.872 | Heptadecanoic acid | | | 27.029 | Pentadecane, 2-methyl- | | | |
| 43.308 | 9,12-Octadecadienoic acid | | | 28.683 | Hexadecane, 4-methyl- | | | |
| 43.445 | 9-Octadecenoic acid | | | 31.172 | Decane, 3,6-dimethyl- | | | |
| 43.594 | 11-Octadecenoic acid | | | 32.053 | Tetradecane, 2,6,10-trimethyl- | | | |
| 48.023 | Stearic acid | | | 32.322 | Heptadecane | | | |
| 51.828 | Behenic acid | | | 34.765 | Hexadecane, 2,6,10,14-tetramethyl- | | | |
| 56.732 | Lignoceric acid | | | 45.334 | Tetracosane | | | |
| 9.463 | Formamide, N,N-diethyl- | | amides | 45.414 | 2-methyloctacosane | | | |
| 10.676 | Acetamide, N,N-diethyl- | | | 52.972 | Docosane | | | |
| 47.496 | Oleamide | | | 58.546 | Hentriacontane | | | |
| 20.489 | 3,5-Dimethylphenyl isothiocyanate | | esters | 61.492 | Heneicosane | | | |
| 37.123 | Benzenepropanoic acid,3,5-bis(1,1-dimethylethyl)-4-hydroxy-,methyl ester | | | 25.616 | Phenol, 2,4-bis(1,1-dimethylethyl)- | | | phenols |
| 37.58 | Dibutyl phthalate | | | 37.185 | 4-Coumaric acid | | | |
| 50.529 | 2-Palmitoylglycerol | | | 18.44 | Nonane, 2-methyl-5-propyl- | | | others |
| 51.221 | 1-Monopalmitin | | | 19.316 | Oxalic acid, 6-ethyloct-3-yl hexyl ester | | | |
| 17.588 | Diethylene glycol | | alcohols | 17.931 | Decane, 1-iodo- | | | |
| 18.795 | Glycerol | |  | 24.82 | Tridecane, 1-iodo- | | | |
| 42.164 | 1-Octadecanol | | | 39.995 | Disulfide, di-tert-dodecyl | | | |
| 36.075 | Pentadecanenitrile | | nitriles | 43.211 | 1-Eicosene | | | |
| 40.933 | Heptadecanenitrile | | | 9.789 | Boric acid | | | |
| 16.472 | Benzaldehyde, 3,5-dimethyl- | | aldehydes | 19.121 | Methoxyacetic acid, 3-tetradecyl ester | | | |
| 32.642 | 3,5-di-tert-Butyl-4-hydroxybenzaldehyde | | | 18.652 | Phosphoric acid |  | | |
| 37.781 | Dulcitol | | sugars |  |  |  | | |
| 53.253 | Sucrose | |  |  |  |  | | |
| 55.982 | D-Trehalose | | |  |  |  | | |
